# The Effects of Statin Dose, Lipophilicity, and Combination of Statins plus Ezetimibe on Circulating Oxidized Low-Density Lipoprotein Levels: A Systematic Review and Meta-Analysis of Randomized Controlled Trials

**DOI:** 10.1155/2021/9661752

**Published:** 2021-09-04

**Authors:** Tannaz Jamialahmadi, Fatemeh Baratzadeh, Željko Reiner, Luis E. Simental-Mendía, Suowen Xu, Andrey V. Susekov, Raul D. Santos, Amirhossein Sahebkar

**Affiliations:** ^1^Department of Food Science and Technology, Quchan Branch, Islamic Azad University, Quchan, Iran; ^2^Department of Nutrition, Faculty of Medicine, Mashhad University of Medical Sciences, Mashhad, Iran; ^3^School of Pharmacy, Mashhad University of Medical Sciences, Mashhad, Iran; ^4^Department of Internal Medicine, University Hospital Centre Zagreb, School of Medicine, University of Zagreb, Zagreb, Croatia; ^5^Biomedical Research Unit, Mexican Social Security Institute, Durango, Mexico; ^6^Division of Life Sciences and Medicine, University of Science and Technology of China, Hefei, China; ^7^GBOU DPO Russian Medical Academy for Postgraduate Medical Education Ministry of Health, Moscow, Russia; ^8^Lipid Clinic Heart Institute (Incor), University of São Paulo, Medical School Hospital, São Paulo, Brazil; ^9^Applied Biomedical Research Center, Mashhad University of Medical Sciences, Mashhad, Iran; ^10^Biotechnology Research Center, Pharmaceutical Technology Institute, Mashhad University of Medical Sciences, Mashhad, Iran

## Abstract

**Background:**

Elevated plasma low-density lipoprotein cholesterol (LDL-C) is the main risk factor for atherosclerotic cardiovascular disease (ASCVD). Statins are the drugs of choice for decreasing LDL-C and are used for the prevention and management of ASCVD. Guidelines recommend that subjects with high and very high ASCVD risk should be treated with high-intensity statins or a combination of high-intensity statins and ezetimibe. The lipophilicity or hydrophilicity (solubility) of statins is considered to be important for at least some of their LDL-C lowering independent pleiotropic effects. Oxidative modification of LDL (ox-LDL) is considered to be the most important atherogenic modification of LDL and is supposed to play a crucial role in atherogenesis and ASCVD outcomes.

**Objective:**

The aim of this systematic review and meta-analysis was to find out what are the effects of statin intensity, lipophilicity, and combination of statins plus ezetimibe on ox-LDL.

**Methods:**

PubMed, Scopus, Embase, and Web of Science were searched from inception to February 5, 2021, for randomized controlled trials (RCTs). Two independent and blinded authors evaluated eligibility by screening the titles and abstracts of the studies. Risk of bias in the studies included in this meta-analysis was evaluated according to the Cochrane instructions. Meta-analysis was performed using Comprehensive Meta-Analysis (CMA) V2 software. Evaluation of funnel plot, Begg's rank correlation, and Egger's weighted regression tests were used to assess the presence of publication bias.

**Results:**

Among the 1427 published studies identified by a systematic databases search, 20 RCTs were finally included in the systematic review and meta-analysis. A total of 1874 patients are included in this meta-analysis. This meta-analysis suggests that high-intensity statin treatment is associated with a significant decrease in circulating concentrations of ox-LDL when compared with low-to-moderate treatment (SMD: -0.675, 95% CI: -0.994, -0.357, *p* < 0.001; *I*^2^: 55.93%). There was no difference concerning ox-LDL concentration between treatments with hydrophilic and lipophilic statins (SMD: -0.129, 95% CI: -0.330, -0.071, *p* = 0.206; *I*^2^: 45.3%), but there was a significant reduction in circulating concentrations of ox-LDL associated with statin plus ezetimibe combination therapy when compared with statin monotherapy (SMD: -0.220, 95% CI: -0.369, -0.071, *p* = 0.004; *I*^2^: 0%).

**Conclusion:**

High-dose statin or combination of statins with ezetmibe reduces plasma ox-LDL in comparison low-to-moderate intensity statin therapy alone. Statin lipophilicity is not associated with reduction in ox-LDL plasma concentrations.

## 1. Introduction

It has been well known for many decades that elevated plasma level of low-density lipoprotein cholesterol (LDL-C) is the most important risk factor for atherosclerotic cardiovascular disease (ASCVD) [1, 2]. For almost four decades, statins are the drugs of choice for treating elevated LDL-C and are used for primary and secondary prevention of ASCVD [3, 4]. Besides, these drugs have been identified to exert several pleiotropic effects relevant to cardiovascular health and improvement of noncardiovascular diseases [5–10]. Both the 2019 European Society of Cardiology (ESC)/European Atherosclerosis Society (EAS) Guidelines for the Management of Dyslipidemias and 2018 American Heart Association (AHA)/American College of Cardiology (ACC) Multisociety Guideline on the Management of Blood Cholesterol recommend that patients with ASCVD, severe hypercholesterolemia, familial hypercholesterolemia, or diabetes should be treated aggressively with high-intensity statins or a combination of high-intensity statins and ezetimibe in order to achieve improved ASCVD outcomes [11, 12]. High-intensity statins were defined as atorvastatin 40-80 mg/day or rosuvastatin 20-40 mg/day. The results of some most recently published studies have confirmed such an approach [13–15]. Unfortunately, most of these high and very-high risk patients in real life do not receive high-intensity lipid lowering therapy [4, 16].

Lipophilic statins include atorvastatin, simvastatin, lovastatin, fluvastatin, cerivastatin, and pitavastatin, while hydrophilic statins include rosuvastatin and pravastatin [17]. The lipophilicity or hydrophilicity of statins is important for their pharmacokinetics and pharmacodynamics, and these characteristics are considered to be crucial for at least some of their LDL-C lowering independent pleiotropic effects [18]. Statin lipophilicity might be important because of its association with hepatoselectivity since lipophilic statins undergo oxidative biotransformation by the CYP450 in hepatocytes and therefore are susceptible to drug-drug interactions and might passively and nonselectively pass through the membranes of nonhepatic tissues thus theoretically having a possible role in some adverse effects of statins, e.g., myopathy [19]. On the other hand, hydrophilic statins employ carrier-mediated mechanisms for uptake, which could reduce their ability to have non-lipid-lowering pleiotropic effects on extrahepatic tissues, and they are excreted largely in an unchanged form [17]. However, in randomized trials and in real-world clinical setting, these differences concerning the effects of statins on myopathy risk have not been proven [20].

Oxidative modification of LDL particles (ox-LDL) is for more than a quarter of century considered to be the most important atherogenic modification of LDL and is supposed to play a crucial role in atherogenesis and ASCVD outcomes [21–23]. This is not only due to the role of ox-LDL in atherosclerotic plaque formation [24] but also because ox-LDL participate in destabilization of the existing atherosclerotic plaques by inducing matrix degradation, fissuring of the plaque and thrombus formation on this site thus causing clinical manifestations such as myocardial infarction (MI) and unstable angina [25].

Nevertheless, there are not many studies analyzing the possible association of statin lipophilicity with plasma ox-LDL concentration neither; it is clear whether there is any association between statin intensity and plasma ox-LDL concentration. Furthermore, the association between the uses of ezetimibe, a cholesterol absorption inhibitor, and plasma ox-LDL is not totally set. Apparently, ezetimibe could protect against the oxidative stress induced by ox-LDL [26]. However, in animal experiments, no significant correlations between atherosclerotic plaque areas and serum concentrations of ox-LDL were proven [27].

Therefore, the aim of this systematic review and meta-analysis was to dissect the effects of statins intensity, lipophilicity, and combination of statins plus ezetimibe on plasma ox-LDL.

## 2. Methods

### 2.1. Search Strategy

This systematic review and meta-analysis was designed according to the 2009 guidelines preferred reporting items for systematic reviews and meta-analysis (PRISMA) statement guidelines [28]. PubMed, Scopus, Embase, and Web of Science were searched from inception to February 5^th^ using the following keywords in titles and abstracts (also in combination with MESH terms): (“Hydroxymethylglutaryl-CoA Reductase Inhibitors” OR simvastatin OR rosuvastatin OR atorvastatin OR pravastatin OR pitavastatin OR mevastatin OR fluvastatin OR lovastatin) AND (“oxidized low density lipoprotein” OR “oxidized LDL” OR OxLDL OR ox-LDL OR “oxidized Low-Density Lipoprotein” OR “minimally modified oxidized-LDL” OR MM-LDL OR MMLDL OR “malondialdehyde-low density lipoprotein” OR “malondialdehyde low density lipoprotein” OR MDA-LDL OR MDALDL OR “MDA-LDL IgM” OR “MDA-LDL IgG” OR “autoantibodies against oxidized low-density lipoprotein” OR “autoantibodies against oxidized low density lipoprotein” OR AuAb-oxLDL OR “antibodies against oxidized LDL” OR Anti-oxLDL).

### 2.2. Study Selection

Clinical studies were included if they met the following inclusion criteria: (i) randomized controlled trial with either parallel or cross-over design, (ii) the studies which investigated the impact of statin intensity (i.e., high- versus low-to-moderate-intensity statin), (iii) statin lipophilicity (lipophilic versus hydrophilic statins) or (iv) adding ezetimibe to statin therapy versus statin monotherapy on plasma ox-LDL concentrations, and (v) presentation of sufficient information at baseline and at the end of follow-up in each group or studies providing the net change values. The exclusion criteria are as follows: (i) nonrandomized trials, (ii) uncontrolled trials, (iii) observational studies with case-control, cross-sectional, or cohort design, (iv) noncomparative studies with statins versus a neutral arm, and (iv) lack of sufficient information at baseline or follow-up.

### 2.3. ox-LDL Assay Methods

In most of the included studies, serum ox-LDL was measured using enzyme-linked immunosorbent assay (ELISA) methods. Three studies used Mercodia ox-LDL kit (Mercodia, Uppsala, Sweden) [29–31], two studies used Mercodia, Inc. kit (Winston-Salem, North Carolina, USA) [32, 33], two studies used SRL kit (Tokyo, Japan) [34, 35], one study used R&D Systems Inc. kit (Minneapolis, Minnesota, USA) [36], one study used Immundiagnostik kit (Bensheim, Germany) [37], three studies used Kyowa Medex MX kit (Kyowa Medex, Inc., Tokyo) [38–40], one study used Daiichi kit (Sekisui Medical, Tokyo, Japan) [41], two studies used Biomedica kit (Wien, Austria) [42, 43], one study used TPI Corporation kit (Johnson City, TN) [44], and four studies did not mention the methods used or assay kits [45–48].

### 2.4. Data Extraction

After removal of duplicate studies, two independent and blinded authors (FB and TJ) evaluated eligibility by screening of the titles and abstracts of the studies. Full reports of the potentially eligible studies were then obtained and evaluated. Any disagreements were resolved by discussion with a third author (AS) until reaching a consensus. Eligible studies were reviewed, and the following data were abstracted: (1) the name of first author, (2) year of publication, (3) study design, (4) type of statin used in the study, (5) dose of statin, (6) treatment duration, (7) patients characteristics, and (8) plasma ox-LDL concentrations.

#### 2.4.1. Quality Assessment

Risk of bias in the studies included in this meta-analysis was evaluated according to the Cochrane instruction [28]. Selection bias, performance bias, attrition bias, detection bias, reporting bias, and other sources of bias were estimated to be high, low, or unclear in each of the included studies.

### 2.5. Quantitative Data Synthesis

Meta-analysis was performed using the Comprehensive Meta-Analysis (CMA) V2 software (Biostat, NJ) [49]. Information regarding sample size, means, and standard deviations from each group were extracted to calculate standardized mean differences (SMDs). We applied SMD because of the different metrics used to assay and report plasma ox-LDL values. Effect size was calculated as follows: (measure at the end of follow‐up in the treatment group − measure at baseline in the treatment group) − (measure at the end of follow‐up in the control group − measure at baseline in the control group). A random effects model (using DerSimonian-Laird method) and the generic inverse variance weighting method were used to compensate for the heterogeneity of studies in terms of study design, treatment duration, and the characteristics of the studied populations [50]. If the outcome measures were reported in median and range (or 95% confidence interval (CI)), mean and SD values were estimated using the method described by Hozo et al. [51]. Where only the standard error of the mean (SEM) was reported, SD was estimated using the following formula: SD = SEM × sqrt (*n*), where *n* is the number of subjects. Effect sizes were expressed as standard mean difference (SMD) and 95% CI. In order to evaluate the influence of each study on the overall effect size, a sensitivity analysis was conducted using the leave-one-out method (i.e., removing one study each time and repeating the analysis) [52, 53].

#### 2.5.1. Metaregression

LDL changes were entered into a random effects metaregression model to explore their association with the estimated effect size.

#### 2.5.2. Publication Bias

Evaluation of funnel plot, Begg's rank correlation, and Egger's weighted regression tests were used to assess the presence of publication bias in the meta-analysis. When there was an evidence of funnel plot asymmetry, potentially missing studies were imputed using the “trim and fill” method [54].

#### 2.5.3. Results

**Figure 1:**
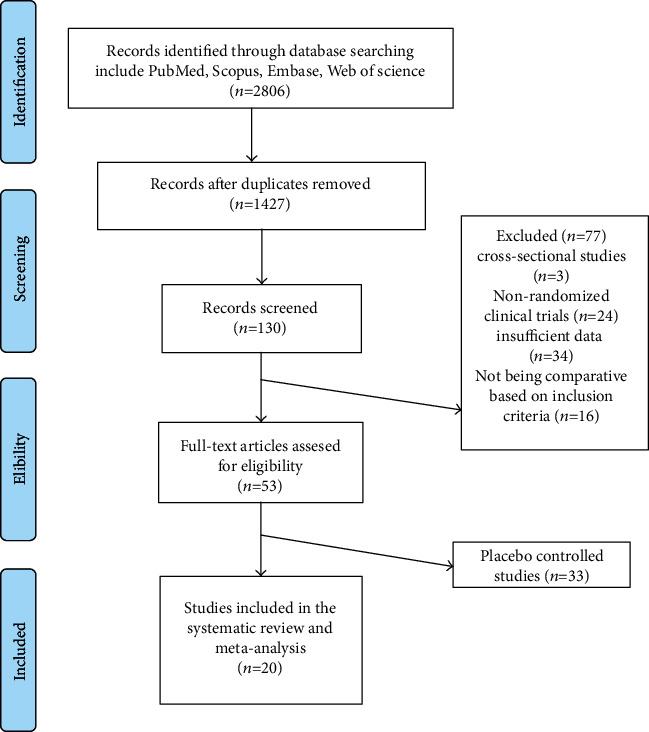
Flow chart of the number of studies identified and included into the meta-analysis.

Among the 1427 published studies identified by a systematic databases search, 130 were found to be potentially relevant following assessment of titles and abstracts. Of those, 77 studies were excluded after careful evaluation (3 studies were cross-sectional, 24 studies were nonrandomized clinical trials, 34 studies did not report sufficient data, and 16 studies were not comparative according to inclusion criteria). However, from remaining 53 studies , 33 studies were not placebo-controlled. Therefore, 20 RCTs were finally included in the systematic review and meta-analysis (Table 1). The study selection process is shown in Figure 1.

## 3. Results

### 3.1. Effects of Statin Intensity on Circulating Concentrations of ox-LDL

**Figure 2:**
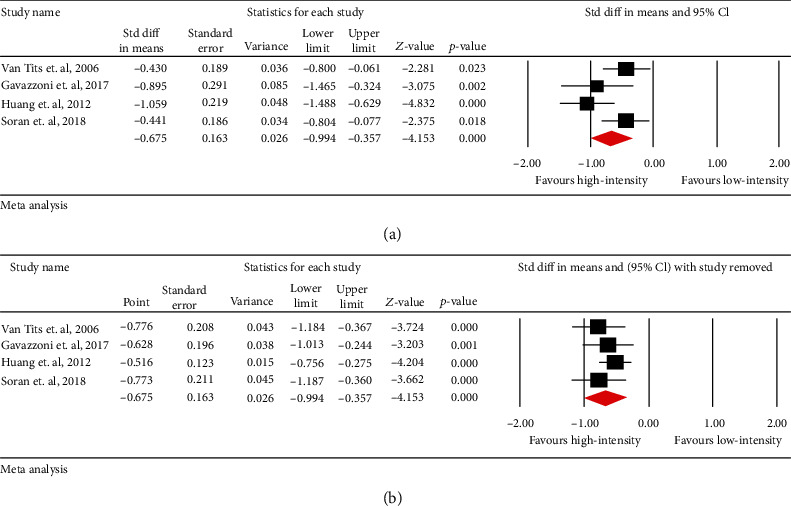
(a) Forest plot displaying standardized mean difference and 95% confidence intervals for the impact of high-intensity statin treatments on circulating concentrations of oxidized LDL. (b) Leave-one-out sensitivity analyses for the impact of high-intensity statin treatment on circulating concentrations of oxidized LDL.

Meta-analysis of data from 4 trials including 381 patients suggested a significant decrease in circulating concentrations of ox-LDL after high-intensity statin treatment vs. low-to-moderate intensity (SMD: -0.675, 95% CI: -0.994, -0.357, *p* < 0.001; *I*^2^: 55.93%) (Figure 2(a)). The reduction in circulating concentrations of ox-LDL was robust in the leave-one-out sensitivity analysis (Figure 2(b)).

### 3.2. Effects of Statin Lipophilicity on Circulating Concentrations of ox-LDL

**Figure 3:**
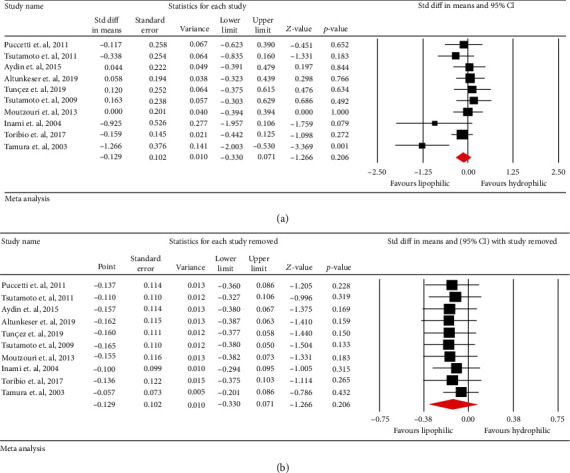
(a) Forest plot displaying standardized mean difference and 95% confidence intervals for the impact of lipophilic statin treatments on oxidized LDL. (b) Leave-one-out sensitivity analyses for the impact of lipophilic statin treatments on circulating concentrations of oxidized LDL.

Meta-analysis of data from 10 trials including 795 patients showed that plasma ox-LDL (SMD: -0.129, 95% CI: -0.330, -0.071, *p* = 0.206; *I*^2^: 45.3%) levels were not significantly different between patients treated with hydrophilic and lipophilic statins (Figure 3(a)). This finding was robust in the leave-one-out sensitivity analysis (Figure 3(b)).

### 3.3. Effects of Statin/Ezetimibe Combination Therapy vs. Statin Monotherapy on Circulating Concentrations of ox-LDL

**Figure 4:**
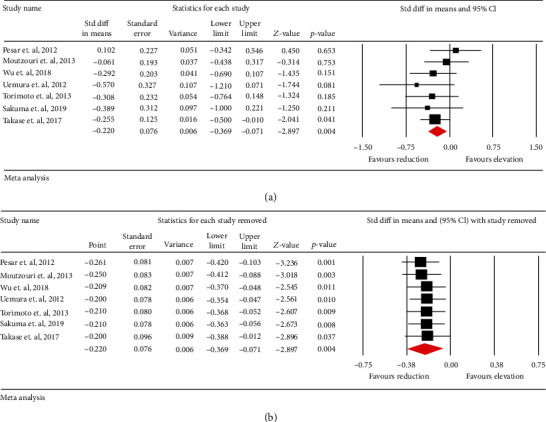
(a) Forest plot displaying standardized mean difference and 95% confidence intervals for the impact of statin/ezetimibe combinational therapy vs statin monotherapy on oxidized LDL. (b) Leave-one-out sensitivity analyses for the impact of statin/ezetimibe combinational therapy vs statin monotherapy on circulating concentrations of oxidized LDL.

Meta-analysis of data from 7 trials including 698 patients suggested a significant reduction in circulating concentrations of ox-LDL following statin/ezetimibe combination therapy vs. statin monotherapy (SMD: -0.220, 95% CI: -0.369, -0.071, *p* = 0.004; *I*^2^: 0%) (Figure 4(a)). The reduction in circulating concentrations of ox-LDL with statin/ezetimibe combination therapy was robust in the leave-one-out sensitivity analysis (Figure 4(b)).

#### 3.3.1. Metaregression

**Figure 5:**
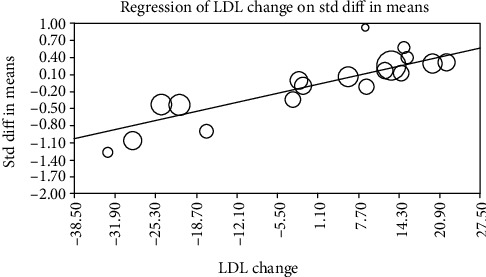
Random effects metaregression for assessing the effect of delta LDL.

Random effects metaregression was performed to assess the impact of changes in LDL concentration on the circulating concentrations of ox-LDL lowering activity of statins. The results suggested a significant association between the changes in circulating concentrations of ox-LDL and delta LDL (slope: 0.024; 95% CI: 0.017, 0.030; *p* < 0.001) (Figure 5).

#### 3.3.2. Publication Bias

**Figure 6:**
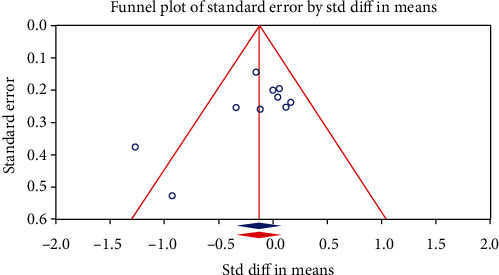
Funnel plot detailing publication bias in the studies reporting the impact of statin lipophilicity on circulating concentrations of oxidized LDL.

Considering the inclusion of 10 RCTs in the meta-analysis of statin lipophilicity, publication bias was assessed using funnel plot. The funnel plot on the association between statin lipophilicity and plasma ox-LDL levels is shown in Figure 6. Although the funnel plot was asymmetric on visual inspection, the “trim and fill” method did not suggest any potentially missing study indicative of publication bias. Moreover, Egger's linear regression test (intercept = −2.38, standard error = 1.28; 95%CI = −5.35, −0.57, *t* = 1.85, df = 8, two-tailed *p* = 0.1) and Begg's rank correlation test (Kendall's Tau with continuity correction = −0.31, *z* = 1.25, two-tailed *p* value = 0.21) did not suggest any publication bias.

## 4. Discussion

Findings of this meta-analysis indicate that high-intensity statin treatment is associated with a significant decrease in circulating concentrations of ox-LDL when compared with low-to-moderate treatment. There were no differences concerning ox-LDL concentrations between treatments with hydrophilic and lipophilic statins, but there was a significant reduction in circulating concentrations of ox-LDL associated with statin plus ezetimibe combination therapy when compared with statin monotherapy. We also explored the association between magnitudes of changes in plasma ox-LDL and LDL-C levels in the studies included in meta-analysis using random effects metaregression. Overall, a significant association was found between alterations in plasma ox-LDL and LDL-C concentrations. Delayed clearance of LDL in hypercholesterolemic people increases the likelihood for circulating LDL to be changed, contributing to the onset and progression of atherosclerosis [55].

ox-LDL is implicated on the pathophysiology of atherosclerosis, and there is evidence that its plasma levels are associated with the risk of ASCVD events [56]. Indeed, plasma ox-LDL correlates positively with LDL-cholesterol concentrations [57]. Therefore, it is reasonable to infer that reduction in the latter would affect plasma ox-LDL. Some studies had shown that statin treatment decreases ox-LDL levels [58–60]. However, to the best of our knowledge, so far no meta-analysis has been published showing whether there are any differences between high-intensity statin treatment and low-to-moderate treatment on ox-LDL levels. In an early study when normolipemic patients were treated with a high dose of atorvastatin, this resulted in a decrease of autoantibodies against ox-LDL [61]. More recently, it has been shown that high-dose atorvastatin and rosuvastatin caused similar decreases in ox-LDL levels [42]. Nevertheless, it has to be mentioned that in a small study on hemodialysis patients on a low dose of a less potent statin (simvastatin 20 mg/day), a significant decrease of ox-LDL also occurred, but in this study, the result was not compared with a higher dose of a more potent statin [62]. The results of this meta-analysis indicate that high-intensity statin treatment is associated with a significant decrease in circulating concentrations of ox-LDL when compared with low-to-moderate treatment may be of clinical importance. Indeed, there is indisputable evidence that high-intensity statin therapy not only further reduces LDL-cholesterol but also prevents more ASCVD events in comparison with low-dose statins [63]. It is conceivable that greater reductions in ox-LDL may could have contributed to that.

There is evidence suggesting that statin solubility could have played a role in the reduction in ASCVD events in patients with diabetes or those undergoing chronic dialysis [64, 65]. However, studies did not present the results of the effects of lipophilic and hydrophilic statins on ox-LDL. Previous studies suggested that there were no differences concerning ox-LDL concentrations between treatments with lipophilic and hydrophilic statins. Our meta-analysis confirms and expands these previous findings. Indeed, benefits of statin therapy on ASCVD are in theory related to their potency in reducing plasma LDL-cholesterol rather than aspects pertaining to statin solubility in water or lipids [63].

One important finding of this meta-analysis was that the combination of statin with ezetimibe was associated with greater reductions in circulating concentrations of ox-LDL when compared with statin monotherapy. It has been shown in the IMPROVE-IT study that a combination of simvastatin a statin plus ezetimibe decreased the risk of ACVD events better in comparison with simvastatin monotherapy. In that study, the benefit was proportional to LDL-cholesterol reduction [66]. However, no meta-analysis has been performed to ascertain whether such a combination therapy could have a beneficial effect on ox-LDL [65–67]. Considering the role that ox-LDL plays in atherogenesis, the findings of this study are intuitively in agreement with those of IMPROVE-IT; however, similar to high-dose statin if further ox-LDL lowering prevents ASCVD events needs to be proven.

Despite including apparently the most adequate studies in the literature with a low risk of bias and the homogeneity of study results, this meta-analysis has some limitations: (1) the meta-analysis of data on the effects of statin intensity on circulating concentrations of ox-LDL included only four trials with 381 patients and therefore these results should be interpreted with caution; (2) although in most of the included studies serum ox-LDL was measured using enzyme-linked immunosorbent assay (ELISA) method, in some studies, other assays/kits were used; and (3) individual rather than study level data would have provided more robust results.

## 5. Conclusions

Based on the results of this meta-analysis, it could be concluded that high-intensity statin treatment is associated with a significant decrease in circulating concentrations of ox-LDL when compared with low-to-moderate statin treatment. There was no difference concerning ox-LDL concentration between treatments with hydrophilic and lipophilic statins, but there was a significant reduction in circulating concentrations of ox-LDL associated with statin plus ezetimibe combination therapy when compared with statin monotherapy. These findings should have mechanistic implications to explain the additional benefits of high-intensity and statin ezetimibe combination in comparison with low-to-moderate-statin therapy alone when prevention of ASCVD events is concerned.

## Figures and Tables

**Table 1:** Characteristics of studies measuring circulating concentrations of oxidized LDL.

Study, year	Study design	Follow-up	Treatment	Control	Clinical outcome		Patients	No. of patients
					ox-LDL	MDA-LDL		
van Tits et al., 2006 [29]	Randomized double-blind study	2 years	A 80 mg/day	S 40 mg/day	Significant decrease in plasma level of ox-LDL	—	Patients with heterozygous familial hypercholesterolemia	115
Puccetti et al., 2011 [45]	Prospective, randomized, double-blind study	8 weeks	A 20 mg/day	R 10 mg/day	Significant decrease in plasma level of ox-LDL	—	Hypercholesterolemic patients	60
Tsutamoto et al., 2011 [38]	Randomized double-blind open-label study	6 months	A 5 mg/day	R 2.5 mg/day	Significant decrease in plasma level of ox-LDL in the A group but not in the R group	—	CHF patients with dilated cardiomyopathy	63
Aydin et al., 2015 [37]	Randomized active-controlled study	4 weeks	A 80 mg/day	R 20 mg/day	Significant decrease in plasma level of ox-LDL	—	STEMI	90
Altunkeser et al., 2019 [42]	Randomized active-controlled study	4 weeks	A 80 mg/day	R 40 mg/day	Significant decrease in plasma level of ox-LDL	—	ACS	106
Tunçez et al., 2019 [43]	Randomized active-controlled study	4 weeks	A 80 mg/day	R 40 mg/day	Significant decrease in plasma level of ox-LDL	—	AMI	63
Gavazzoni et al., 2017 [36]	Single-center, prospective, randomized, blinded open-label study	1 month	A 80 mg/day	A 20 mg/day	Significant decrease in plasma level of ox-LDL	—	STEMI	52
Soran et al., 2018 [31]	Double-blind randomized study	12 months	A 80 mg/day	A 10 mg/day	Significant decrease in plasma level of ox-LDL	—	T2DM with microalbuminuria or proteinuria	119
Huang et al., 2012 [44]	Double-blind randomized study	12 months	A 40 mg/day	A 10 mg/day	Significant decrease in plasma level of ox-LDL	—	Patients with ischemic cardiomyopathy	95
Tamura et al., 2003 [34]	Randomized crossover study	8 weeks	A 10 mg/day	P 10 mg/day	—	Significant decrease in plasma level of MDA-LDL	Hypercholesterolemic patients	34
Tsutamoto et al., 2009 [39]	Randomized active-controlled study	4 months	S 5 mg/day	R 2.5 mg/day	Significant decrease in plasma level of ox-LDL in the R group but not in the S group	—	Patients with nonischaemic CHF	71
Moutzouri et al., 2013 [30]	Prospective, randomized, open-label, blinded endpoint study	12 weeks	S 40 mg/day	R 10 mg/day	Significant decrease in plasma level of ox-LDL	—	Hypercholesterolemic patients	100
				S 10 mg/day + Ez 10 mg/day	Significant decrease in plasma level of ox-LDL		Hypercholesterolemic patients	108
Inami et al., 2004 [40]	Prospective, randomized active-controlled study	12 weeks	P 10 mg/day	F 20 mg/day	Significant decrease in plasma level of ox-LDL	—	Hypercholesterolemic patients	16
Toribio et al., 2017 [32]	Double-blind, active-controlled, parallel-group comparative study	52 weeks	Pi 4 mg/day	P 40 mg/day	Significant decrease in plasma level of ox-LDL	—	HIV-infected patients with dyslipidemia	192
Pesaro et al., 2012 [33]	Randomized, double-blind, active-controlled study	6 weeks	S 80 mg/day	S 20 mg/day + Ez 10 mg/day	Significant decrease in plasma level of ox-LDL	—	CAD	78
Uemura et al., 2012 [35]	Randomized open-label crossover study	12 weeks	A 20 mg/day	A 10 mg/day + Ez 10 mg/day	—	Decrease in plasma level of MDA-LDL	Abnormal glucose tolerance and CAD	39
Wu et al., 2018 [46]	Randomized active-controlled study	12 weeks	A 40 mg/day	A 20 mg/day + Ez 10 mg/day	Significant decrease in plasma level of ox-LDL	—	ASCVD	98
Torimoto et al., 2013 [41]	Randomized open-label study	12 weeks	R 5 mg/day	R 2.5 mg/day + Ez 10 mg/day	—	Significant decrease in plasma level of MDA-LDL	T2DM	75
Sakuma et al., 2019 [47]	Randomized crossover study	3 months	Double dose of statin	Ez 10 mg + baseline dose of statin	—	Significant decrease in plasma level of MDA-LDL	CAD	42
Takase et al., 2017 [48]	Multicenter, prospective, randomized, open-label, blinded-end point study	6-8 months	Statin monotherapy	Ez 10 mg/d + statin	—	Significant change in plasma level of MDA-LDL	CAD patients after coronary stenting	258

A: atorvastatin; ASCVD: atherosclerotic cardiovascular disease; ACS: acute coronary syndrome; AMI: acute myocardial infarction; CAD: coronary artery disease; CHF: Congestive heart failure; EZ: ezetimibe; F: fluvastatin; MDA-LDL: malondialdehyde modified low-density lipoprotein; ox-LDL: oxidized low-density lipoprotein; P: pravastatin; Pi: pitavastatin; R: rosuvastatin; S: simvastatin; T2DM: type 2 diabetes.

## Data Availability

There is no primary raw data associated with this review article.
